# Potential antiaging activity of secretome gel of human Wharton’s jelly mesenchymal stem cells (hWJ-MSCs) in UV-induced mice models

**DOI:** 10.22038/IJBMS.2024.70825.15385

**Published:** 2024

**Authors:** Wahyu Widowati, Ahmad Faried, Achmad Adam, Deni Rahmat, Hanna Sari Widya Kusuma, Nindia Salsabila Mia Dewi, Marisca Evalina Gondokesumo, Rizal Rizal, Ita Margaretha Nainggolan, Massoud Vosough

**Affiliations:** 1 Faculty of Medicine, Maranatha Christian University, Bandung, West Java, Indonesia; 2 Department of Neurosurgery, Faculty of Medicine, Universitas Padjadjaran-Dr. Hasan Sadikin Hospital, Bandung, West Java, Indonesia; 3 Oncology and Stem Cell Working Group, Faculty of Medicine, Universitas Padjadjaran-Dr. Hasan Sadikin Hospital, Bandung, West Java, Indonesia; 4 Faculty of Pharmacy, Pancasila University, South Jakarta, Indonesia; 5 Biomolecular and Biomedical Research Center, Aretha Medika Utama, Bandung, West Java, Indonesia; 6 Faculty of Pharmacy, University of Surabaya, Universitas Surabaya, Surabaya, East Java, Indonesia; 7 Biomedical Engineering Department of Electrical Engineering, Faculty of Engineering University of Indonesia, Jakarta, Indonesia; 8 Clinical Pathology Department, School of Medicine and Health Sciences, Atma Jaya Catholic University, Jakarta, Indonesia; 9 Eijkman Research Center for Molecular Biology, National Research and Innovation Agency, Bogor, West Java, Indonesia; 10 Cell Science Research Center, Royan Institute for Stem Cell Biology and Technology, Tehran, Iran; 11 Experimental Cancer Medicine, Institution for Laboratory Medicine, Karolinska, Institute, Stockholm, Sweden

**Keywords:** Inflammation, Mesenchymal stem cells, Oxidative stress, Secretome, Skin aging

## Abstract

**Objective(s):**

Skin aging is a degenerative process that can be induced by UV irradiation. UV radiation can produce reactive oxidate stress which causes premature aging. This study aims to examine the antiaging potential of secretome gel (SC) from human Wharton Jelly Mesenchymal Stem Cells (hWJ-MSCs) in a UVB-induced mice model.

**Materials and Methods:**

The secretome was obtained from hWJ-MSCs and made in gel form. Male mice were radiated by UVB for 15 min twice daily for 14 days. The gel was topically applied to the mice’s dorsal skin. Two treatments of secretome gel: secretome 1 is applied once and secretome 2 is applied twice daily after UVB radiation. TGF-β1, IL-10, and IL-18 gene expression was determined using RT-PCR. Hematoxylin Eosin staining was used to observe the inflammation and collagen density of skin tissue. An immunohistochemistry assay was used to analyze the protein expression of P53, COL4A1, MMP-2, and MMP-13. The data were statistically analyzed using the ANOVA test followed by the Tukey post hoc test (*P*<0.05).

**Results:**

UVB induction caused loss of collagen, increasing inflammation and high expression of aging mediators. SC increased the gene expression of TGF-β1 and IL-10 and decreased IL-18 gene expression. Histopathological tests showed that SG increased collagen density, lowered inflammation, and repaired cell damage in skin tissue. Immunohistochemistry test showed that SC decreased MMP-2, MMP-13, and P53 expression, in contrast, increased COL4A1.

**Conclusion:**

The secretome gel of hWJ-MSCs showed antiaging activities with potential for preventing and curing skin aging.

## Introduction

The skin serves as a physical barrier between the body and the outside environment. In addition to preventing water loss and microbial infection, it serves an essential cosmetic function (1). Therefore, the skin is the first part of the body to be exposed to various exposures from the outside environment, making it susceptible to tissue damage, particularly skin aging. Skin aging is a multisystem degenerative process that affects both the skin and the skin support system. Skin aging can be induced by a variety of causes, including UV irradiation, stress, and smoking (2). Up to 80% of the accelerated skin aging process can be related to excessive ultraviolet (UV) exposure, often known as skin photoaging (3). UV is a critical component of sunshine. Two types of UV harm the skin, namely UVB (290–320 nm), UVA (320–400 nm), and UVC (100-280 nm) (4). UVB and UVA can induce the formation of Reactive Oxidative Stress (ROS), which cause damage to DNA, proteins, and lipids. Direct UVB absorption by DNA leads to molecular rearrangements that result in the formation of certain photoproducts, like cyclobutane dimers and 6-4 photoproducts. In contrast, UVA can generate ROS through indirect photosensitizing reactions. Many of these DNA changes have the potential to cause mutations and cancer (5).

The signs of aging skin include wrinkles, elasticity loss, laxity, and an appearance of rough texture. In addition to structural and functional changes in extracellular matrix elements like collagen and elastin, this aging process is accompanied by phenotypic alterations in skin cells (1). The length of time exposure to UV radiation from sunlight can determine damage to the skin. Sunburn, inflammation, immunological suppression, and dermal connective tissue damage result from acute UV irradiation of human skin (2). Recent studies conducted over the last few decades have demonstrated that UV exposure causes oxidative stress due to excessive formation of ROS (6, 7).

ROS as free radicals play an important role in skin aging and dermal damage process (8). Oxidative stress promotes signaling transduction pathways for Mitogen-Activated Protein Kinase (MAPK). Multiple immunosuppressive and proinflammatory cytokines such as Interleukin-18 (IL-18) can be released by MAPK, which is followed by an increase in the production of Matrix Metalloproteinases (MMPs) (9, 10). MMPs play a crucial role in the invasion of tumors, inflammatory response, and skin aging (11–13). In particular, MMP-2 and MMP-13 are involved in skin aging which can degrade interstitial collagen fibrils (14). Besides MMPs, P53 is an important inducer of aging. A study conducted by Kim *et al*. (2014) reported that mice with prolonged P53 activation exhibit an aging phenotype in the skin, with decreased subcutaneous fat and sebaceous gland atrophy (15). Another study found that Mouse Double Minute 2 Homolog (MDM2) depletion, as the main negative regulator of P53, generated an aging phenotype in mouse skin, including epidermal thinning, decreased wound healing, and gradual fur loss (16). In contrast, Transforming Growth Factor β-1 (TGFβ-1) is a key regulator of Extracellular Matrix (ECM) protein synthesis in human skin, stimulating fibroblast proliferation and collagen formation (17). Thus, inhibition of P53 activity and increased expression of Collagen-4A1 (COL-4A1)and TGFβ-1 is one way to prevent photoaging due to free radicals from UV exposure. Therefore, development of more effective therapies must be found.

Stem cell-based therapies are still being developed today, particularly in the treatment of skin diseases. Stem cells, especially Mesenchymal Stem Cells (MSCs), can release trophic substances that are crucial for tissue regeneration and repair (18). However, this MSC therapy has low cell survival and engraftment rates after the transplant process, cell retention, and, in the case of stem cell alterations, can lead to cancer (19, 20). MSCs and other stem cells secreted a bioactive substance in a conditioned medium called secretome. The secretome comprises a variety of macromolecules, extracellular vesicles, including microvesicles and exosomes, cytokines, growth factors, and other substances that can stimulate a variety of biological processes, particularly in the modulation of the development of diverse new tissues (21–23). Numerous studies have reported the ability of secretome to regenerate and repair damaged tissues, which can be one of the options for treating numerous skin diseases, particularly skin aging.

In general, people use sunblock to protect the skin from the side effects of UV radiation. The composition of the active substances contained in a sunblock product can determine its effectiveness in protecting the skin from UV radiation. In our study, we used secretome from hWJ-MSCs as the main active ingredient gel of our treatment for photoaged mice skin. Various growth factors contained in the secretome and made in gel form are expected to inhibit various proteins that trigger aging and accelerate skin tissue regeneration. Therefore, we investigated the effect of secretome gels of hWJ-MSCs as an antiaging agent in UVB-induced photoaging in mice by measuring TGF-β1, IL-10, IL-18 gene expression, P53, COL-4A1, MMP-2, and MMP-13 protein expression, and expression of inflammation and collagen in the skin tissue.

## Materials and Methods

***Secretome gel preparation***

The secretome was obtained from human Wharton’s Jelly Mesenchymal Stem Cells (hWJ-MSCs) culture. A total of 20 ml secretome was mixed with 5 g of polyvinyl pyrrolidone (PVP) (Himedia, MB102-0100) and 5 g of lactose. Then the mixture was freeze-dried and made into powder. The basis gel formulation was prepared in two processes. Gel base 1: 2 g Carboxy Methyl Cellulose (CMC) (Sigma, 419273-100G) in 100 ml of water, while gel base 2 : 5 gr of triethanolamine (Glent, GE8879-1L) was added dropwise to 2 g of carbopol 940 (Maestro Kimia) in 95 ml of water. Base gels 1 and 2 were mixed until homogenous. A total of 0.2% (w/w) Na benzoate, 1% (w/w) Lecithin (Himedia, GRM637-0100), L-cysteine 0.5% (w/w) (Himedia, GRM883-0100), 10 % (w/w) polyethylene glycol (PEG) 400 (Subur Kimia Jaya) were added gradually to the mixture of gel and mixed homogeneously. Afterward, 10 g of freeze-dried secretome powder was added into the mixture of gel base and mixed homogeneously. For vehicle control gel group treatment, the mixture of gel base was used (24).

***Animal experimental design***

Twenty-five male mice (*Mus musculus* L.) DDY strains aged 10–11 weeks, weighing from 30–35 grams, were purchased from iRATco Veterinary Laboratory Services Bogor, Indonesia. Mice were acclimatized for a week to adapt to the environment. Mice were kept in individually ventilated cages with controlled temperature (20–24 °C) and relative humidity of 55% for 12 hr each day and night that complied with the Humane Use and Care of Laboratory Animals’ guidelines (25). Mice were fed *ad libitum* using a standard basal metabolism diet (14% protein, 5% fat, PT Indofeed) (26). UV exposure was used to induce aging in mice’s skin. The dorsal of the mice’s hair was shaved to expose UV directly to the mice’s skin (27). The UVB radiation was carried out using Kernel brand UVB light, (KN-4003) for 15 min twice (08:00–08:15 am and 1:00–1:15 pm) daily for 14 days. The secretome gel, sunblock (3W Clinic Intensive UV SPF50+ PA+++), and mixed basis gel are given topically applied to dorsal mice skin at 08:15 am and 1:15 pm (2). The animal experiment procedure is carried out under the direction of the Animal Ethics iRATco VLS No.4.2.003-1/KEHI/IX/2022. The mice were then randomly divided into six groups with four replications. Negative control group (NC): untreated mice. Positive control group (PC) shaved and UVB-induced mice. In vehicle control groups (VC): positive control that was applied with base gel. Comparative Control (CC): positive control applied with sunblock. Secretome gel 1 group (SC1): positive control and applied with gel secretome once application daily at 08.15 am. Secretome gel 2 group (SC2): positive control and applied with gel secretome twice daily at 08.15 am and 1.15 pm.

***Sample collection***

Termination in mice was carried out at 15 days by cervical dislocation (26). The dorsal area of the mice’s skin was excised with a diameter of 3 cm, then the skin was taken and stored in 10 ml of 10% formaldehyde solution before analysis (28).

***Determination of TGF-β1, IL-10 and IL-18 gene expression using RT-PCR***

mRNAs were extracted and purified using the Direct-zol RNA Miniprep Plus (Zymo, R2073). Meridian Bioscience, BIO-65053 for RT-PCR was used to convert the mRNAs into cDNAs (Meridian Bioscience, BIO-65053). According to the manufacturer’s instructions, qPCR was carried out using SYBR NO-ROX kit (Meridian Bioscience, BIO-98005). Real-Time PCR AriaMX 3000 (Agilent, G8830A) was used to analyze the quantitative levels of gene expression. At 260/280 nm, each sample’s RNA concentration and purity were assessed (Table 1). The primer sequences are shown in Table 2. The pre-incubation cycle for this system was set at 95 °C for 5 min, followed by 40 cycles of denaturation at 95 °C for 30 sec, followed by annealing for each gene, which was TGF- β1, IL-10, and IL-18, at 57 °C with 40 cycles. Pre-elongated and elongated were carried out for one minute at 72 °C (26, 29). 

***Hispatology assay***

Hematoxylin and eosin (HE) staining was used to conduct histopathological examination. The skin organs were placed in a formalin solution. Fixation with 10% formalin for 2-3 days followed by dehydration with ascending graded alcohol for 2 hr and then cleaning with alcohol using graded xylol while continuing to shake. Then the organs were embedded into liquid paraffin in stages up to a paraffin concentration of 100% at 60 °C. Then they were stored until paraffin blocks were formed at room temperature. The paraffin block was then sliced using a microtome (Leica RM 2135 BioCut Rotary Microtome) with a thickness of 5 µM. The organ sheets were then put on glass objects and stained using HE. The slides were analyzed using a light microscope and ImageJ software for quantification of the score inflammation, and collagen density (6, 30).

***Immunohistochemistry (IHC) assay***

The skin tissue slices ± 5 mm were de-paraffinized, re-hydrated, and washed in cold tap water before antigen retrieval. Antigen retrieval (Elabscience, E-IR-R213) was performed at 121 °C for 10 min in citrate buffer (pH 6.0) followed by endogenous blocking using 3% H_2_O_2_ (Elabscience, E-IR-217C) in methanol (Merck, 106009) for 15 min at room temperature (31). P53 polyclonal antibody (Elabscience, E-AB-32468), COL-4A1 polyclonal antibody (Elabscience, E-AB-22150), MMP-2 polyclonal antibody (Elabscience, E-AB-32054), and MMP-13 polyclonal antibody (Elabscience,E-AB-70107) as primary antibodies were incubated at room temperature overnight. The Rabbit-Specific HRP/DAB (ABC) Detection IHC Kit was used (Elabscience, E-IR-R213). Haematoxylin was used as a counterstain. The primostar (Zeiss) microscope and a lumenera infinity 1-3c were used to photograph the stained skin tissues. The quantification method of scoring used ImageJ software to assess indices of positive cells on IHC slides (26, 30).

***Statistical analysis***

Software SPSS version 20.0 was used to analyze the data (IBM Corp., Armonk, NY, USA). One-way ANOVA was followed by Tukey *post hoc* used for data interpretation with *P*<0.05 considered to be significant. The result was then presented with the mean ± standard deviation (32).

## Results

***Effect of secretome gel on TGF- β1*** ***gene expression***

**Figure 1:**
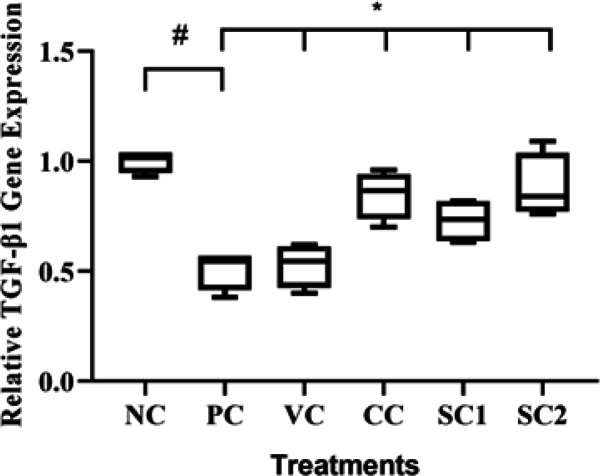
Effect of secretome gel towards TGF-β1 gene expression in aging mice model

The effect of SC on TGF-β1 gene expression in an aging mice model can be seen in Figure 1. UV-induced mice (PC) showed a very significant decrease in TGF-β1 expression compared to NC. Treatment with SC with twice topical application at 08.15 am and 1.15 pm significantly increased the relative expression of the TGF-β1 gene (0.88) in the aging mice model compared to the PC group (0.51) (*P*<0.05). Treatment with SC with twice topical application (SC2) was more effective compared to the once application (Figure 1).

***Effect of secretome gel on IL-10 gene expression***

**Figure 2:**
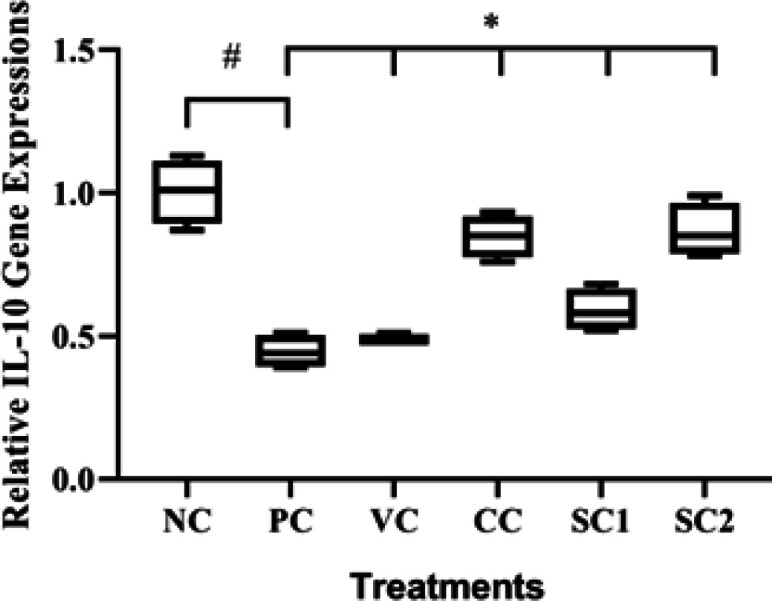
Effect of secretome gel towards IL-10 gene expression in aging mice model

The effect of SC on IL-10 gene expression in an aging animal model can be seen in Figure 2. The PC group has significantly decreased relative IL-10 gene expression (0.45) compared to NC (1.00) (*P*<0.05). Treatment with SC2 significantly increased the relative IL-10 gene expression (0.87) in the aging mice model compared to PC (*P*<0.05). The SC2 treatment was comparable with CC (Figure 2).

***Effect of secretome gel on IL-18 gene expression***

**Figure 3:**
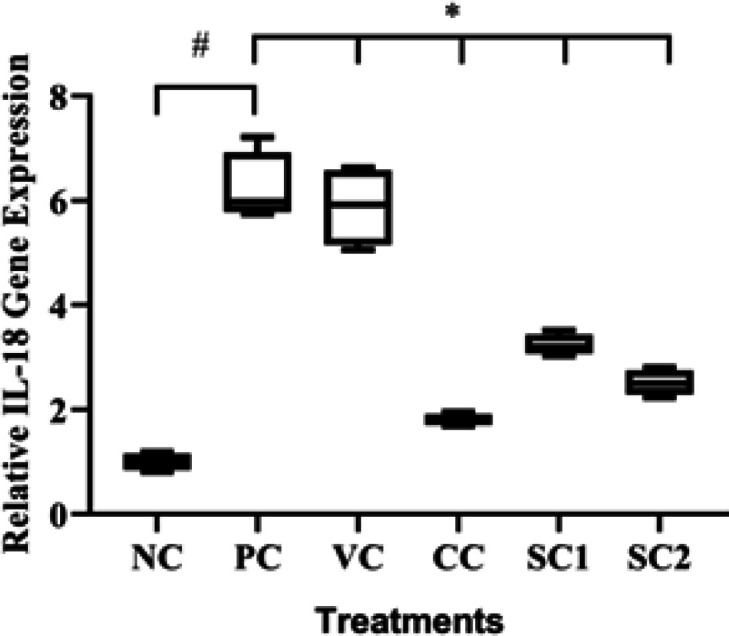
Effect of secretome gel towards IL-18 gene expression in aging mice model

The effect of secretome gel on IL-18 gene expression in an aging mice model can be seen in Figure 3. PC significantly increased relative IL-18 expression compared to NC. The SC treatments were found to significantly reduce relative IL-18 expression (3.23 for SC1 and 2.51 for SC2) compared to PC (6.23) (*P*<0.05), although the effectiveness is lower than CC (1.80). The SC2 treatment was more effective compared to the SC1 treatment.

***Effect of secretome gel on skin tissue histopathology***

**Figure 4:**
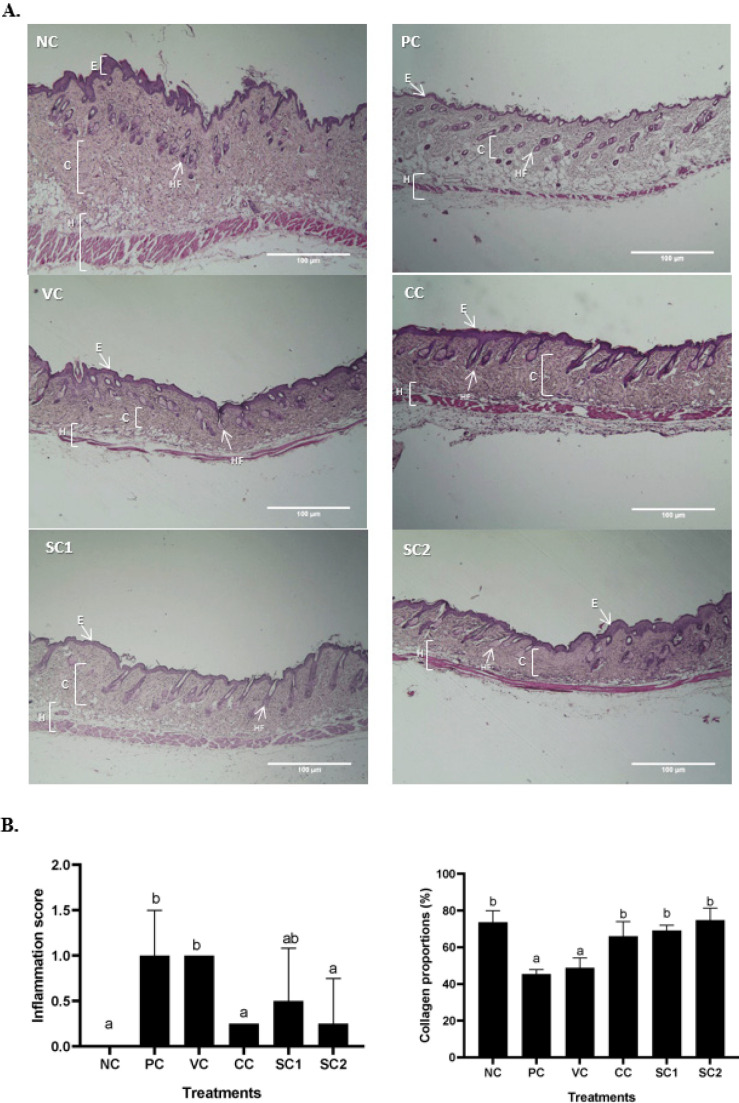
Effect of secretome gel towards inflammation score, collagen density in aging mice model

The histopathology results showed that UVB treatment damaged collagen density and induced inflammation in the skin (Figure 4A). Based on Figure 4A, the epidermis and hypodermis of mice skin in the PC group were thickened compared to other groups. The collagen structure was also damaged in the PC group, meanwhile in SC and CC (sunblock SPF50) groups the collagen structure improved after UVB exposure. The density of collagen was scored (Figure 4B), and the results showed that the collagen proportion areas of PC and VC (basis gel) decreased significantly compared to NC. Meanwhile, there was no significant difference in collagen density between NC, CC, and SC (*P*<0.05), with the highest collagen density found in SC2 groups (74.75%). The inflammation score (Figure 4B) also showed that SC1 and SC2 had a lower score (0.50 and 0.25) respectively compared to the PC group (1.00) and CC (sun block) group (0.25).

***Effect of secretome gel on COL-4A1 protein expression***

**Figure 5:**
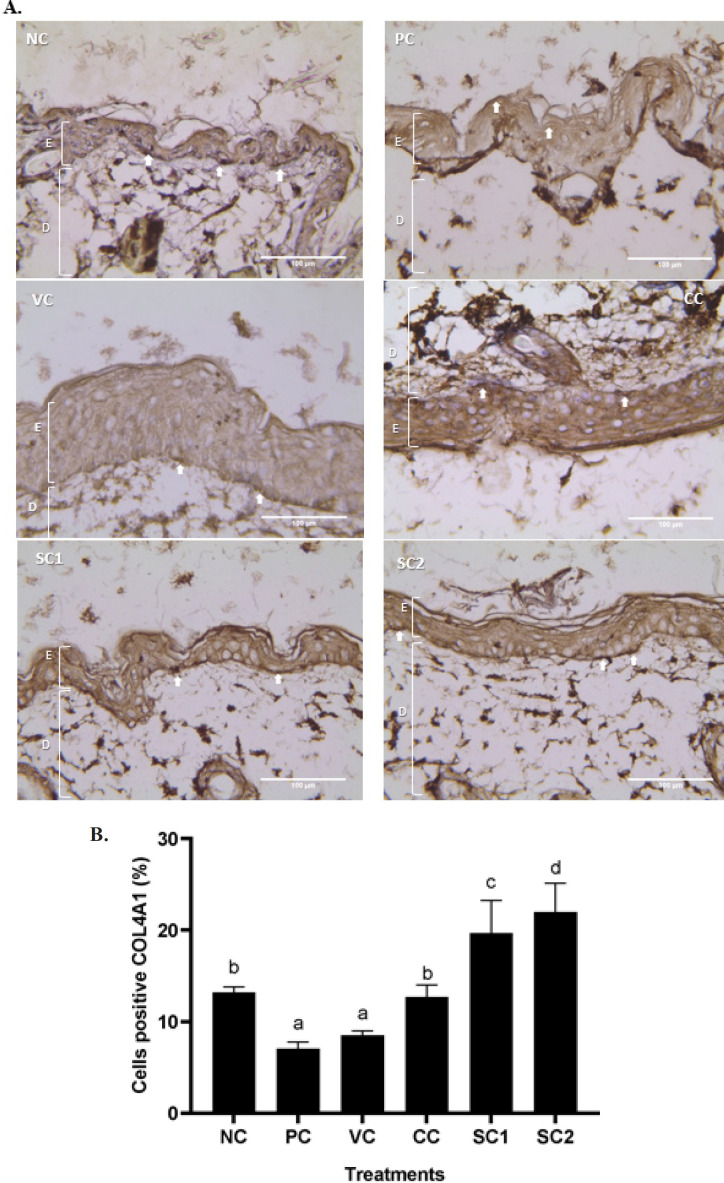
**. **Effect of secretome gel towards COL-4A1 protein expression in aging mice model

Figure 5A-B shows an evaluation of the SC effect on COL-4A1 expression in an aging mice model. COL-4A1 expression is distinguishable by brown cytoplasmic staining (red arrows) and hematoxylin counter. The results showed that UV induction has the capacity to decrease the COL-4A1 protein expression significantly compared to NC (*P*<0.05) (Figure 5B), this result is in line with IHC analysis which showed that only a slight brown color appears. Meanwhile, both SC1 and SC2 could increase COL-4A1 expression. The highest expression of COL-4A1 was found in SC2 compared to CC and VC.

***Effect of secretome gel on MMP-2 protein expression***

**Figure 6:**
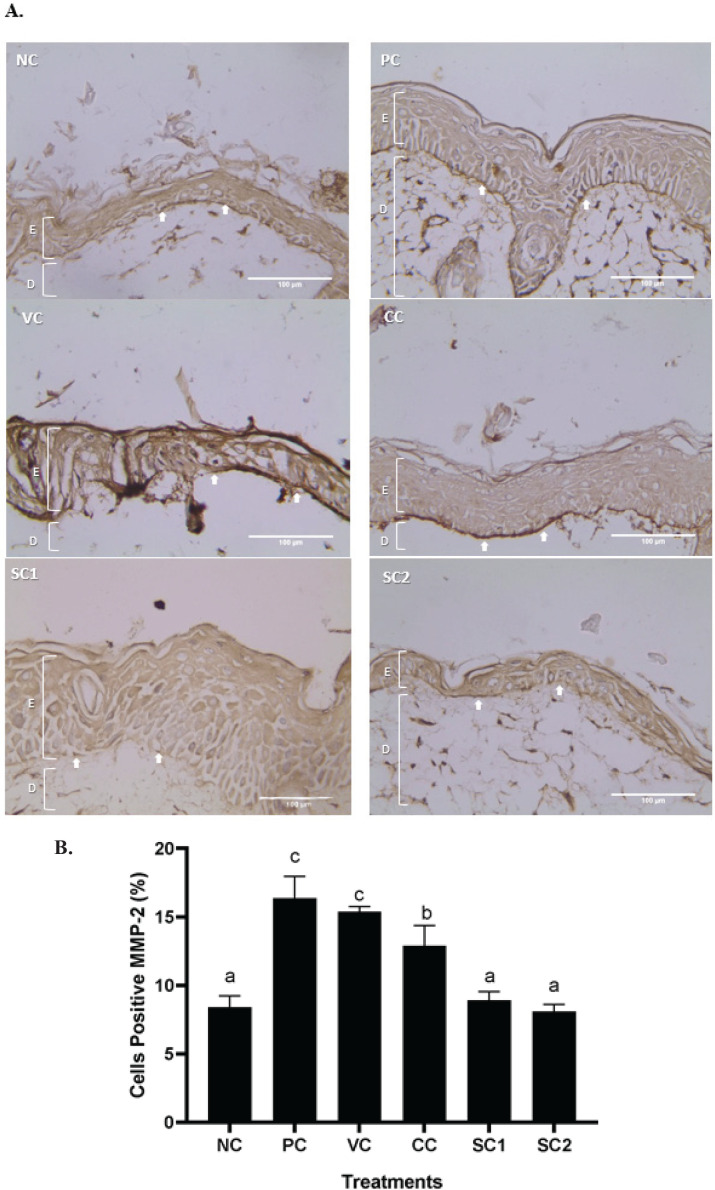
Effect of secretome gel towards MMP-2 protein expression in aging mice model

The results of an IHC assay showed that the skin treated with secretome gel is rejuvenated. The presence of MMP-2 protein was shown by the brown color. A darker tint implies a higher MMP-2 expression, which is a sign of skin injury showed in PC and VC. The secretome gel treatments 1 and 2 had a lower intensity of the brown color (light brown), indicating that the secretome gel could down-regulate MMP-2 expression (Figure 6A). Calculation score of MMP-2 protein expression showed that SC1 and SC2 reduced MMP-2 protein expression (Figure 6B). There was no significant difference between NC (8.41%), SC1 (8.90%) and SC2 (8.10) treatments. The highest score of MMP-2 protein expression value was found in the PC group.

***Effect of secretome gel on MMP-13 protein expression in aging mice model***

**Figure 7:**
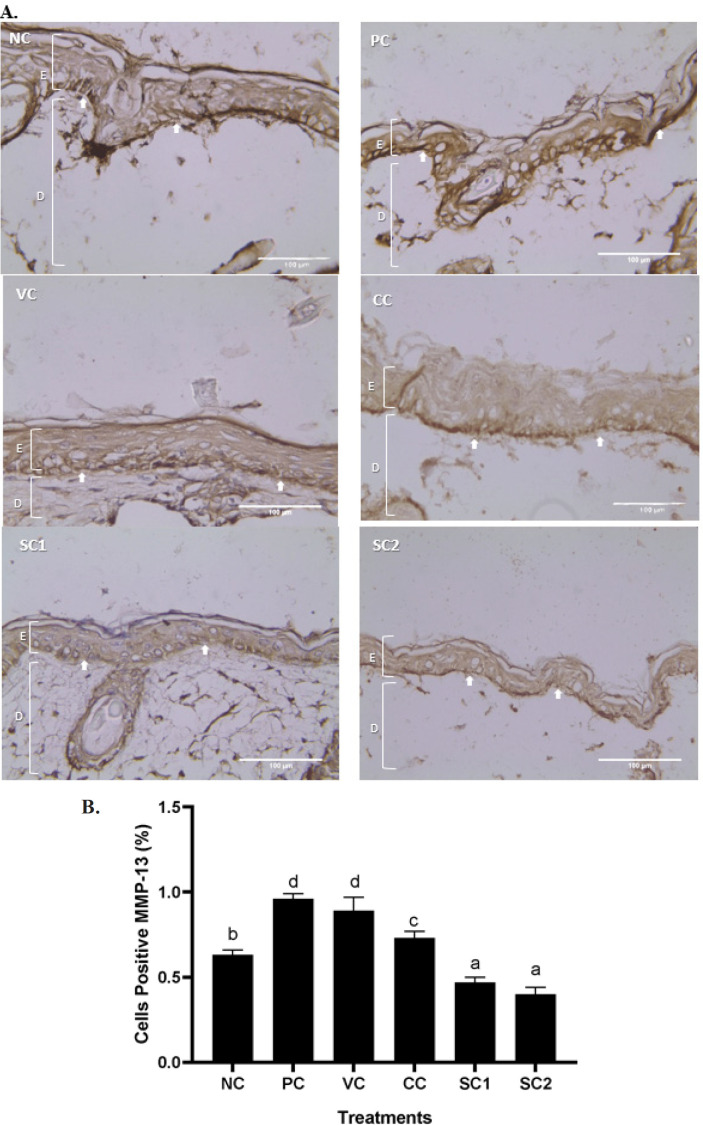
Effect of secretome gel towards MMP-13 protein expression in aging mice model

Figure 7 shows SC effect on MMP-13 protein expression in an aging mice model. Based on Figure 7A both SC1 and SC2 could decrease MMP-13 expression with the best result found in SC2 groups indicated by the brown color appearing in the IHC assays. The results showed that SC1 (0.47%) and SC2 (0.40%) have the capacity to decrease the MMP-13 protein expression significantly compared to PC (*P*<0.05) (Figure 7B). The MMP-13 expression of VC treatment (0.89%) and the PC (0.96%) had no significant differences.

***Effects of secretome gel on P53 protein expression in aging mice model***

**Figure 8:**
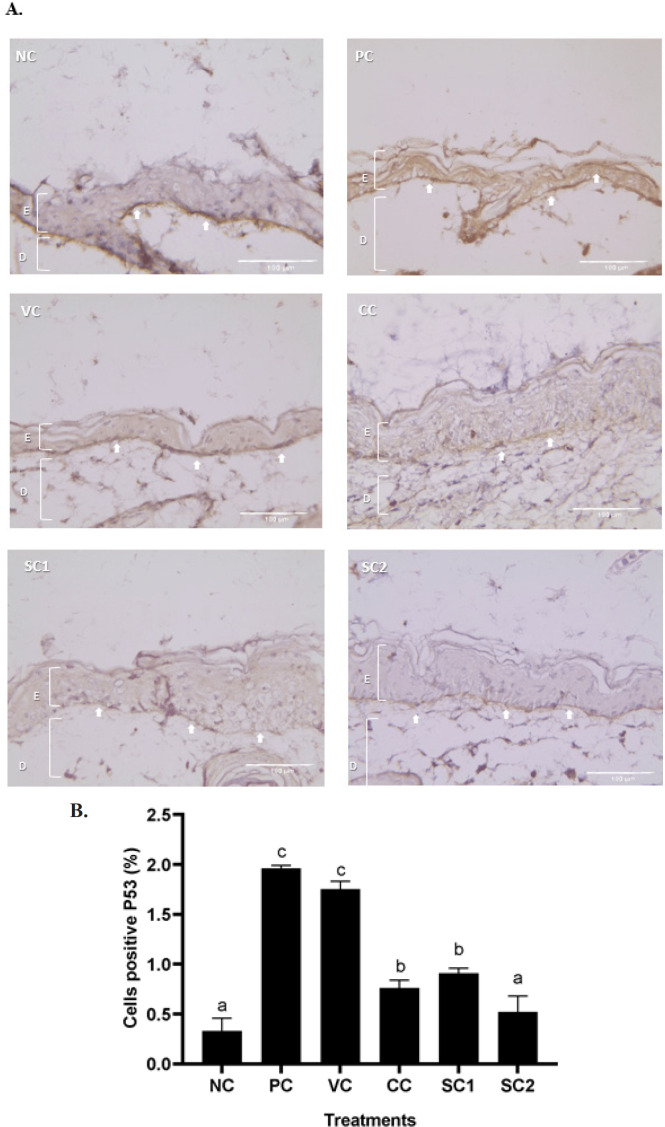
Effect of secretome gel towards P53 protein expression in aging mice model

Based on IHC analysis (Figure 8A), there were dark brown colors with different intensities as a result of P53 protein expression. The brown colors appear the most in the PC group, and the brown color spread until the epidermis structure, compared to the NC and SC groups. The least brown color appears in NC and SC2 groups. The quantification of P53 expression in cells was shown in Figure 8B. The significant differences between PC and NC are indicated by different letters (a, c). In contrast, NC and the SC1 groups have no significant differences. The CC treatment (sunblock SPF50) was not significantly different from SC1 treatment, this result data indicates that SC was comparable with CC to lower P53 expression, and SC2 was the most active to lower P53 expression. 

**Table 1:** Concentration and purity of RNA concentration and purity of each sample

**Treatment**	**RNA concentration** **(ng/ml)**	**RNA purity ** **(λ260/λ280 nm)**
NC (untreated mice)	43.62	2.3966
PC (UV-induced mice)	132.74	2.0694
VC (positive control+base gel)	100.98	2.0752
CC (positive control+sun block)	170.48	2.0686
SC1 (positive control+secretome gel topically 1 time daily) SC2 (positive control+secretome gel topically 2 times daily)	53.42 92.72	2.1034 2.0237

**Table 2.** Primer sequence of mice β-Actin, TGF-β1, IL-18, IL-10

**Gene symbols**	**Primer sequence (5’ - 3’) ** **upper strand: forward** **lower strand: reverse**	**Product size** **(bp)**	**Annealing** **(** ^0^ **C)**	**References**
β-Actin	5’-AAGATCAAGATCATTGCTCCTCC-3’ 5’-TAACAGTCCGCCTAGAAGCA-3’	164	57	NM_007393.5
TGF-β1	5’- AACCAAGGAGACGGAATACAG-3’ 5’- TGGAGCTGAAGCAATAGTTGG-3’	241	60	NM_011577.2
IL-18	5’- GAAGTGATAGCAGTCCCA-3’ 5’- AGCTAAAATCAGCAAAGTGTC-3’	258	58	NM_011339.2
IL-10	5’- GAAGACAATAACTGCACCCA-3’ 5’- AACCCAAGTAACCCTTAAAGTC-3’	163	58	NM_010548.2

* Data are presented as mean ± standard deviation. NC: negative control (untreated mice); PC: positive control (UVB-induced mice); VC: vehicle control (positive control+base gel); CC: comparative control (positive control+sunblock SPF50); SC1: positive control+secretome gel once time daily; SC2: positive control+secretome gel twice times daily. The pound symbol (#) indicates a significant difference between negative control and positive control. An asterisk (*) symbol indicates a significant difference among treatments and the positive control based on the Tukey HSD *post hoc* test (*P*<0.05).

* Data are presented as mean ± standard deviation. NC: negative control (untreated mice); PC: positive control (UVB-induced mice); VC: vehicle control (positive control+base gel); CC: comparative control (positive control+sunblock SPF50); SC1: positive control+secretome gel once daily; SC2: positive control+secretome gel twice daily. The pound symbol (#) indicates a significant difference between negative control and positive control. An asterisk (*) symbol indicates a significant difference among treatments and the positive control based on the Tukey HSD post hoc test (*P*<0.05).

* Data are presented as mean ± standard deviation. NC: negative control (untreated mice); PC: positive control (UVB-induced mice); VC: vehicle control (positive control+base gel); CC: comparative control (positive control+sunblock SPF50); SC1: positive control+secretome gel once daily; SC2: positive control+secretome gel twice daily. The pound symbol (#) indicates a significant difference between negative control and positive control. An asterisk (*) symbol indicates a significant difference among treatments and the positive control based on the Tukey HSD *post hoc* test (*P*<0.05).

(A) Histological Analysis of collagen density in skin tissue. NC: negative control (untreated mice); PC: positive control (UVB-induced mice); VC: vehicle control (positive control+base gel); CC: comparative control (positive control+sunblock SPF50); SC1: positive control+secretome gel once daily; SC2: positive control+secretome gel twice daily. E: Epidermis; C: Collagen; HF: Hair Follicle; H: Hipodermis. In comparison to the normal group and treatment group, the epidermis, dermis, and hipodermis of the positive control group were markedly thicker. (B) Inflammation score and collagen density in UVB-induced mice model. * Data are presented as mean ± standard deviation. The different letters (a,ab, b) significantly differ among treatments toward inflammation score (figure 4A) and different letters (a,b) significantly difference among treatment based on the Tukey HSD *post hoc* test (*P*<0.05).

(A) Immunohistochemistry of COL4A1 protein in skin tissue. *NC: negative control (untreated mice); PC: positive control (UVB-induced mice); VC: vehicle control (positive control+base gel); CC: comparative control (positive control+sunblock SPF50); SC1: positive control+secretome gel once daily; SC2: positive control+secretome gel twice daily. E: Epidermis; D: Dermis; Arrows ( ) indicate the location of protein expression. (B) COL4A1 protein expression in UVB-induced mice model. * Data are presented as mean ± standard deviation. The different letters (a,b,c,d) significantly difference among treatments based on the Tukey HSD *post hoc* test (*P*<0.05).

(A) Immunohistochemistry of MMP-2 protein in skin tissue. (B) Percentage MMP-2 protein expression in UVB-induced mice model *NC: negative control (untreated mice); PC: positive control (UVB-induced mice); VC: vehicle control (positive control+base gel); CC: comparative control (positive control+sunblock SPF50); SC1: positive control+secretome gel once daily; SC2: positive control+secretome gel twice daily. E: Epidermis; D: Dermis; Arrows ( ) indicate the location of protein expression. (B) MMP-2 protein expression in UVB-induced mice model. *Data are presented as mean ± standard deviation. The different letters (a,b,c) significantly difference among treatments based on the Tukey HSD *post hoc* test (*P*<0.05).

(A) Immunohistochemistry of MMP-13 protein in skin tissue. (B) Percentage MMP-13 protein expression in UVB-induced mice model. *Data are presented as mean ± standard deviation. *NC: negative control (untreated mice); PC: positive control (UVB-induced mice); VC: vehicle control (positive control+base gel); CC: comparative control (positive control+sunblock SPF50); SC1: positive control+secretome gel once daily; SC2: positive control+secretome gel twice daily. E: Epidermis; D: Dermis; Arrows ( ) indicate the location of protein expression. Different letters showed a significant difference among treatments based on the Tukey HSD *post hoc* test (*P*<0.05).

A) Immunohistochemistry of P53 protein in skin tissue. (B) Percentage P53 protein expression in UVB-induced mice model. *Data are presented as mean ± standard deviation. *NC: negative control (untreated mice); PC: positive control (UVB-induced mice); VC: vehicle control (positive control+base gel); CC: comparative control (positive control+sunblock SPF50); SC1: positive control+secretome gel once daily; SC2: positive control+secretome gel twice daily. E: Epidermis; D: Dermis; Arrows ( ) indicate the location of protein expression. (B) P53 protein expression in UVB-induced mice model. *Data are presented as mean ± standard deviation. Different letters showed a significant difference among treatments based on the Tukey HSD *post hoc* test (*P*<0.05).

## Discussion

Skin is continuously exposed to many hazardous environmental stimuli, causing the skin to experience premature aging. There are two types of aging processes (intrinsic and extrinsic) that take place simultaneously. Extrinsic aging, also known as photoaging, is more common than skin cancer and is mostly caused by UV radiation (33). UV radiation over time causes the ongoing breakdown of ECM proteins like collagen and elastin, as well as a slower rate of collagen renewal/synthesis. The application of MSCs in the treatment of skin diseases is still being developed today. It has been proposed that alternative measures such as exosomes, microvesicles, and secretomes produced from various MSCs sources can show regenerative effects and could potentially be utilized as a supplement to existing preventive and therapeutic approaches (21). Here, our study highlights the potential effectiveness of the hWJMSCs secretome gel. 

The histological features and alterations in the skin of the mice were examined by staining with H&E and IHC. Histological examination of each slide was done for collagen density and inflammation score in the skin tissue. Meanwhile, IHC was done for detecting cells containing protein COL-4A1, MMP-2, MMP-13, and P53. It was discovered that exposure to UVB rays induced pathological characteristics linked to skin aging.

According to our findings, the mice treated with hWJMSCs secretome gel had a lower inflammatory cytokine (IL-18) (Figure 3) compared to PC, higher anti-inflammatory cytokine (IL-10) (Figure 2), and a higher TGF-β1 expression (Figure 1) for cell growth control, cell proliferation, tissue regeneration compared to PC. This finding is consistent with a previous study that IL-10 and TGF-β1 gene expression were significantly lower both in the epidermis and dermis skin tissue of aging mice model. Another study reported that conditioned medium human adipose tissue mesenchymal stem cells (CM-hATMSCs) lower ROS level as inflammation inducers compared to non-treated with CM-hATMSCs (34). IL-10 and TGF-β1 are acting as anti-inflammatory cytokines and growth factors that induce tissue regeneration. Secretome gel contains growth factors that induce the production of other growth factors in tissues, especially TGF-β1. The results data were in line with the inflammation score by HE examination. The secretome gel lowered inflammation score compared to the PC and was comparable with NC. The inflammation and aging as a result of UV induction are suppressed by the presence of secretome gel, it was shown by the low inflammation score and the proinflammatory gene expressions.

A previous study reported that in mice with athymia, acute disruption of the epidermal permeability barrier causes an increase in cutaneous and serum inflammatory cytokines (35). One of the disruptions of epidermal permeability is the loss of permeability factor, particularly collagen. The loss of collagen can be induced by UV radiation which can lead the skin aging and the loss of permeability (36).

This study revealed that based on the histopathological evaluation there is a decreased collagen density in the PC group compared to CC, and SC in which all groups were radiated with UVB (Figure 4). The highest collagen density was found in SC2 followed by an increase in COL-4A1 protein expression evaluated by IHC. The COL-4A1 gene codes for one component of type IV collagen, a flexible protein that is essential for the construction of various integral component tissues throughout the body (37). Another study reported that UVB radiation reduced the moisture capacity of the animals’ skin. However, the mice treated with Human Mesenchymal Stromal Cells Conditioned Medium (CM-HMSCs) based formulation provided UVB protection (69.92%) and greatly reduced skin moisture loss from UVB exposure (38). The study conducted by Widowati *et al*. (2022) reported that CM-hATMSCs increased the collagen density, viability, and decreased ROS level in human skin fibroblast (BJ) aging cells model treated with H_2_O_2 _(39). 

Transcriptional factor pathways are activated as a result of UV-induced cellular damage to the DNA and molecular levels, which in turn controls the expression of many genes and proteins including MMP-2, MMP-13, and P53. MMP-2, MMP-13, and P53 play a role in the aging process. UV-radiated animals in SC group displayed a substantial decrease in MMP-2, MMP-13, and P53 compared to PC and VC group. The excess production of MMP-2 can degrade several ECM components such as COL type I and IV (27). Another study revealed that the expression of MMP-2 and MMP-9 was much more abundant in aged tenocytes, and gelatin zymography assay demonstrated that MMP-2 and MMP-9 enzymatic activity increased dramatically with age (40). Meanwhile, MMP-13 can degrade fibrillar collagen (41). This study reports that the protein expressions of MMP-13 increase in PC (UVB-radiated). The results are in line with previous research showing that the levels of MMP-3 and MMP-13 mRNA and protein were significantly increased in the UVB-induced skin photoaging in hairless mice (42).

Besides MMP-2 and MMP-13, the P53 protein is also involved in the skin aging process. The study conducted by (15) reported that mice with prolonged P53 activation exhibit an aging phenotype in the skin, with decreased subcutaneous fat and sebaceous gland atrophy (SG). These findings suggested that MMP-2, MMP-13, and P53 proteins are taking an important role in the aging process, and in this study, they can be down-regulated by SC treatment. The secretome contains growth factors and enhances tissue regeneration (43). Another study demonstrated that four weeks of therapy with the secretome of Adipose Stem Cell (ASC) improved photoaged skin via a paracrine mechanism by boosting the thickness of the epidermal and dermal layers, expression of MMP-1 and Tissue Inhibitor of Metalloproteinase-1 (TIMP-1), and the density of the dermal collagen (33). 

Overall, the secretome treatment has the best effect on the animal tests compared to the basis gels even the comparative product. This is due to the biologically active compound of secretome gel. In basic gel, there is no active compound present so in the collagen density, anti-inflammatory cytokine and protein expression associated with anti-aging is lower compared to the secretome group. Secretomes contain bioactive compounds such as a growth factor that enhances the regeneration of skin tissues and reduces inflammation as shown by decreasing the proinflammatory cytokine and elevating anti-inflammatory cytokines or protein expressions.

## Conclusion

hWJ-MSCs secretome gel have antiaging activity by up-regulating TGF-β1 and IL-10 gene expression, increasing the collagen density and COL-4A1 protein expression. In contrast, the secretome gel down-regulate IL-18 gene expression, MMP-2, MMP-3, and P53 protein expression in the UV-induced aging mice model. Therefore, hW-JMSCs secretome gel is able to potentiate as an antiaging agent and is promising for use in skin aging treatment and prevention.

## Authors’ Contribnutions

W W, A F, A A, and D R designed the experiments; W W, D R, HSW K, NS MD, and M V performed experiments and collected data; HSW K, NS MD, and IMN analyzed and interpreted the data; W W, NS MD, ME G, R R, and M V drafted the manuscript; A F, A A, ME G, and R R analyzed the data, and supervised and directed the study; W W, A F, A A, D R, HSW K, NS MD, ME G, R R, IM N, and M V approved the final version to be published.

## Conflicts of Interest

None.
